# Supplementary material for: Glibenclamide, ATP and metformin increases the expression of human bile salt export pump ABCB11
Source: F1000Res. 2020 Dec 22;9:1497. [Version 1] doi: 10.12688/f1000research.26632.1 (PMC7953918; doi:10.12688/f1000research.26632.1)
Supplement: Peer review history

# Peer review: Reviewer response for version 1

**DOI:** 10.5256/f1000research.29399.r79055

**Published:** 2021-03-09

**Authors:** Amit Dash

**Affiliations:** 1 Baylor College of Medicine, Houston, TX, USA

In the article by Vats et al., the authors used the preexisting microarray data set of the liver samples from ABCB11 knockout mice and used different bioinformatics methods to draw a relationship between ABCB11 and other proteins, microRNAs, and drugs. They validated the predicted drugs by real-time PCR and Western blot in HepG2 cells. The authors did an outstanding job in this pilot study which may be helpful for future researchers. Though their approach and methodologies are right but for better reproducibility and understanding, authors need to explain the following points

 Major comments:

1. Why authors choose HepG2 cells and not other hepatoma cell lines? They need to describe it in the discussion.
2. Authors need to give justification about their chosen drug concentration. Is it the optimum concentration?
3. For more reproducibility of the data, it will be nice if authors mention the detailed protein extraction method and whether they used whole-cell extract or any fractions like nuclear and cytoplasmic extract.
4. In figure 7a, the authors need to mention what is on the Y-axis of the graph and need to put error bars and statistical significance. The method of statistical analysis needs to discuss in materials and methods. Also, it will be nice if authors make a graph for the western blot taking the averages and errors of three independent experiments and normalized with actin.
5. From their raw data, it is surprising that the endogenous control (GAPDH or 18S) is highly variable between controls and treatments. Did the authors start with an equal amount of RNA? How much RNA they used for cDNA. Also, it is confusing out of 18S and GAPDH, which they used for endogenous control. If they used 18S, they need to mention the primer sequences.
6. Authors need to recheck the GAPDH primers as the melting point is more than one.

Minor comments:

1. Under introduction, on line “we explored *in silico* **the **interactions/networks around,” **"the"** can be omitted.
2. Fig1 resolution needs to be increased.

Is the work clearly and accurately presented and does it cite the current literature?

Yes

If applicable, is the statistical analysis and its interpretation appropriate?

Partly

Are all the source data underlying the results available to ensure full reproducibility?

Partly

Is the study design appropriate and is the work technically sound?

Yes

Are the conclusions drawn adequately supported by the results?

Yes

Are sufficient details of methods and analysis provided to allow replication by others?

Partly

Reviewer Expertise:

Cancer biology, transcription, nuclear receptor, cell biology

I confirm that I have read this submission and believe that I have an appropriate level of expertise to confirm that it is of an acceptable scientific standard, however I have significant reservations, as outlined above.

# Peer review: Reviewer response for version 1

**DOI:** 10.5256/f1000research.29399.r76680

**Published:** 2021-02-03

**Authors:** Jayanta Roy-Chowdhury

**Affiliations:** 1 Department of Medicine, Montefiore Medical Center, Bronx, NY, USA

In this manuscript the authors have used the data on hepatic gene expression submitted to NCBI by Zhang Y, Neale G and Schuetz to determine the effect on the haplodeficiency of *ABCB11* (BSEP) in mouse liver. The 375 upregulated genes included those involved in cellular transport and innate immunity (e.g. IFN signaling), whereas the 185 downregulated genes included signal transduction proteins, e.g. epidermal growth factor receptor and those involved with metabolic pathways. In addition, they have examined the effect of Glibenclamide, metformin and ATP on the expression of human ABCB11 in the human cell line HepG2. All three chemicals were found to increase ABCB11 expression.

 Comments:

 The information gene pathway analysis provides useful information as it has revealed "nodes" and "hubs" that mediate the up regulation and down regulation of the genes the expression of which is dysregulated in deficiency of ABCB11 function. However, as the original data set had been derived from whole mouse liver, it comprises gene expression by hepatocytes as well as non-parenchymal cells. It is known that bile acid accumulation can affect gene expression in various cell types in addition to hepatocytes (such as lung cells). Perhaps, the authors can mention this in the Discussion section.

 A second concern is that HepG2 cells are not the most appropriate cell line for modeling the induction of ABCB11 in human hepatocytes, because, unlike some other human hepatoma cell lines, these cells lack the most abundant hepatocyte microRNA, miR-122. miR-122 is known to target various genes, thereby enhancing IFN signaling. On the other hand IFN downregulates the expression of miR-122. Thus, transcriptional induction of ABCB11 in HepG2 cell may not parallel that in primary human hepatocytes. Therefore, the drug induction study should be validated in a different human hepatoma cell line, or at least, the authors should discuss this complexity in interpreting the results.

Is the work clearly and accurately presented and does it cite the current literature?

Yes

If applicable, is the statistical analysis and its interpretation appropriate?

Yes

Are all the source data underlying the results available to ensure full reproducibility?

Yes

Is the study design appropriate and is the work technically sound?

Partly

Are the conclusions drawn adequately supported by the results?

Partly

Are sufficient details of methods and analysis provided to allow replication by others?

Yes

Reviewer Expertise:

Hepatology; Genetic disorders; Cell transplantation; Gene therapy

I confirm that I have read this submission and believe that I have an appropriate level of expertise to confirm that it is of an acceptable scientific standard, however I have significant reservations, as outlined above.

# Peer review: Reviewer response for version 1

**DOI:** 10.5256/f1000research.29399.r76410

**Published:** 2021-01-25

**Authors:** Premkumar Kumpati

**Affiliations:** 1 Department of Biomedical Science, Bharathidasan University, Tiruchirappalli, Tamil Nadu, India

The authors have provided an interesting manuscript that described the role of Bile salt export pump (BSEP/ABCB11) and its relationship with other proteins, microRNAs and Drugs. Various bioinformatics tools were used to explore and carried out experiments to validate and correlate their findings.

 Overall, I believe that this manuscript is well written and will provide some useful information for the scientific community. Having said that there are some concerns I have that the authors should address before it is ready/accept for indexing.

****Minor**** ****Comments to the Author****

1. The author can provide more information on biological mechanism of ABCB11.
2. What is the rational for selecting concentration of drugs used glibenclamide (500  ng/mL), metformin (25 mg/L) or ATP (1 mM).
3. What is the basis for selecting Glibenclamide & ATP out of the total hits identified using bioinformatic analysis.
4. Providing Uniport accession number of each gene in table 1 could be more informative, authors may consider doing it.
5. Image quality for Fig.1 & 4 may be increased.

Is the work clearly and accurately presented and does it cite the current literature?

Yes

If applicable, is the statistical analysis and its interpretation appropriate?

Yes

Are all the source data underlying the results available to ensure full reproducibility?

Yes

Is the study design appropriate and is the work technically sound?

Yes

Are the conclusions drawn adequately supported by the results?

Yes

Are sufficient details of methods and analysis provided to allow replication by others?

Yes

Reviewer Expertise:

Genomics, Genetics, Nanotheraputics, Cancer biology

I confirm that I have read this submission and believe that I have an appropriate level of expertise to confirm that it is of an acceptable scientific standard.
